# Supplementary material for: Lifetime depression and age-related changes in body composition, cardiovascular function, grip strength and lung function: sex-specific analyses in the UK Biobank
Source: Aging (Albany NY). 2021 Jul 7;13(13):17038–79. doi: 10.18632/aging.203275 (PMC8312429; doi:10.18632/aging.203275)
Supplement: Supplementary Material 1 [file aging-13-203275-s001.pdf]

Supplementary Material 1. CIDI-SF lifetime depression criteria.

Supplementary Table 1. Case definition lifetime depression.

	Definition and UK Biobank data fields
**Lifetime depression** (case):<br>At least one core symptom of major depressive disorder, most or all of the day on most or all days for a two-week period, with at least five depressive symptoms that represent a change from usual occurring over the same timescale, with some or a lot of impairment.<br><br>No record of psychosis or bipolar disorder.	(“Ever had prolonged feelings of sadness or depression” (20446) = Yes OR “Ever had prolonged loss of interest in normal activities” (20441) = Yes)<br>AND<br>“Fraction of day affected during worst episode of depression” (20436) = Most of day or All day long<br>AND<br>“Frequency of depressed days during worst episode of depression” (20439) = “Almost every day” or “Every day” AND “Impact on normal roles during worst period of depression” (20440) = “Somewhat” or “A lot”<br>AND<br>Total number of symptoms endorsed (core and others) ≥5: “Ever had prolonged feelings of sadness or depression” (core) (20446), “Ever had prolonged loss of interest in normal activities” (core) (20441), “Feelings of tiredness during worst episode of depression” (20449), “Weight change during worst episode of depression” (20536), “Did your sleep change?” (20532), “Difficulty concentrating during worst depression” (20435), “Feelings of worthlessness during worst period of depression” (20450), “Thoughts of death during worst depression” (20437)<br>AND<br>**No** self-reported psychosis or mania for “Mental health problems ever diagnosed by a professional” (20544)<br>AND<br>**No** self-reported mania/bipolar disorder/manic depression or schizophrenia for “Non-cancer illness” (20002)<br>AND<br>**No** ICD-10 code for “manic episode” or “bipolar affective disorder” (F30-F31) or “schizophrenia, schizotypal and delusional disorders” (F20-F29)<br>AND<br>**No** probable bipolar disorder (20126) according to Smith et al. (2013)<br>AND<br>**No** bipolar disorder record according to the MHQ<br>AND<br>**No** primary care record of bipolar disorder or psychosis

Note: Criteria for lifetime depression adapted from Davis et al. (2020), doi: 10.1192/bjo.2019.100. CIDI-SF, Composite International Diagnostic Interview Short Form; ICD-10, International Classification of Diseases, Tenth Revision; MHQ, mental health questionnaire.
