# Supplementary material for: Lifetime depression and age-related changes in body composition, cardiovascular function, grip strength and lung function: sex-specific analyses in the UK Biobank
Source: Aging (Albany NY). 2021 Jul 7;13(13):17038–79. doi: 10.18632/aging.203275 (PMC8312429; doi:10.18632/aging.203275)
Supplement: Supplementary Material 2 [file aging-13-203275-s002.pdf]

Supplementary Material 2. ICD-10 codes for depression.

Depressive episode.

- F32.0 Mild depressive episode.
- F32.1 Moderate depressive episode.
- F32.2 Severe depressive episode without psychotic symptoms.
- F32.3 Severe depressive episode with psychotic symptoms.
- F32.8 Other depressive episodes.
- F32.9 Depressive episode, unspecified.

Recurrent depressive disorder.

- F33.0 Recurrent depressive disorder, current episode mild.
- F33.1 Recurrent depressive disorder, current episode moderate.
- F33.2 Recurrent depressive disorder, current episode severe without psychotic symptoms.
- F33.3 Recurrent depressive disorder, current episode severe with psychotic symptoms.
- F33.4 Recurrent depressive disorder, currently in remission.
- F33.8 Other recurrent depressive disorders.
- F33.9 Recurrent depressive disorder, unspecified.
