# Supplementary material for: Lifetime depression and age-related changes in body composition, cardiovascular function, grip strength and lung function: sex-specific analyses in the UK Biobank
Source: Aging (Albany NY). 2021 Jul 7;13(13):17038–79. doi: 10.18632/aging.203275 (PMC8312429; doi:10.18632/aging.203275)
Supplement: Supplementary Material 3 [file aging-13-203275-s003.pdf]

Supplementary Material 3. Smith et al. depression criteria.

Depression (adapted from Smith et al. (2013), doi: 10.1371/journal.pone.0075362).

3. Single probable episode of major depression:
4598 ever depressed/down for a whole week; plus 4609 at least two weeks duration; plus
4620 only one episode, plus 2090 ever seen a GP or 2100 a psychiatrist for nerves, anxiety, depression
OR
4631 ever anhedonic (unenthusiasm/uninterest) for a whole week; plus 5375 at least two weeks; plus
5386 only one episode; plus 2090 ever seen a GP or 2100 a psychiatrist for nerves, anxiety, depression.

4. Probable recurrent major depression (moderate):
4598 ever depressed/down for a whole week; plus 4609 at least two weeks duration; plus 4620 at least two episodes; plus 2090 ever seen a GP (but not a psychiatrist) for nerves, anxiety, depression
OR
4631 ever anhedonic (unenthusiasm/uninterest) for a whole week; plus 5375 at least two weeks; plus
5386 at least two episodes; plus 2090 ever seen a GP (but not a psychiatrist) for nerves, anxiety, depression.

5. Probable recurrent major depression (severe):
4598 ever depressed/down for a whole week; plus 4609 at least two weeks duration; plus 4620 at least two episodes; plus 2100 ever seen a psychiatrist for nerves, anxiety, depression
OR
4631 ever anhedonic (unenthusiasm/uninterest) for a whole week; plus 5375 at least two weeks; plus
5386 at least two episodes; plus 2100 ever seen a psychiatrist for nerves, anxiety, depression.
