# Supplementary material for: Lifetime depression and age-related changes in body composition, cardiovascular function, grip strength and lung function: sex-specific analyses in the UK Biobank
Source: Aging (Albany NY). 2021 Jul 7;13(13):17038–79. doi: 10.18632/aging.203275 (PMC8312429; doi:10.18632/aging.203275)
Supplement: Supplementary Material 4 [file aging-13-203275-s004.pdf]

Supplementary Material 4. Psychotropic medication codes.

Supplementary Table 2. Psychotropic medication codes.

UK Biobank code	Drug name
1140879616	Amitriptyline
1140921600	Citalopram
1140879540	Fluoxetine
1140867878	Sertraline
1140916282	Venlafaxine
1140909806	Dosulepin
1140867888	Paroxetine
1141152732	Mirtazapine
1141180212	Escitalopram
1140879634	Trazodone
1140867876	Prozac
1140882236	Seroxat
1141190158	Cipralex
1141200564	Duloxetine
1140867726	Lofepramine
1140879620	Clomipramine
1140867818	Nortriptyline
1140879630	Imipramine
1140879628	Dothiepin
1141151946	Cipramil
1140867948	Amitriptyline
1140867624	Prothiaden
1140867756	Trimipramine
1140867884	Lustral
1141151978	Reboxetine
1141152736	Zispin
1141201834	Cymbalta
1140867690	Anafranil
1140867640	Doxepin
1140867920	Moclobemide
1140867850	Phenelzine
1140879544	Fluvoxamine
1141200570	Yentreve
1140867934	Triptafen
1140867758	Surmontil
1140867914	Tranylcypromine
1140867820	Allegron
1141151982	Edronax
1140882244	Molipaxin
1140879556	Mianserin
1140867852	Nardil
1140867860	Faverin
1140917460	Nefazodone
1140867938	Amitriptyline+Chlordiazepoxide
1140867856	Isocarboxazid
1140867922	Manerix
1140910820	Maoi
1140882312	Sinequan
1140867944	Tranylcypromine+Trifluoperazine
1140867784	Ludiomil
1140867812	Norval
1140867668	Tryptizol
1140867940*	Fluphenazine hydrochloride+Nortriptyline 1.5mg/30mg tablet
1140867942*	Fluphenazine hcl+Nortriptyline 500micrograms/10mg tablet
1140928916	Olanzapine
1141152848	Quetiapine
1140867444	Risperidone

1140879658	Chlorpromazine
1140868120	Trifluoperazine
1141153490	Amisulpride
1140867304	Sulpiride
1141152860	Seroquel
1140867168	Haloperidol
1141195974	Aripiprazole
1140867244	Stelazine
1140867152	Depixol
1140909800	Flupentixol
1140867420	Clozapine
1140879746	Promazine
1141177762	Risperdal
1140867456	Modecate
1140867952	Fluanxol
1140867150	Flupenthixol
1141167976	Zyprexa
1140882100	Zuclopenthixol
1140867342	Clopixol
1140863416	Largactil
1141202024	Abilify
1140882098	Fluphenazine
1140867184	Haldol
1140867092	Serenace
1140882320	Clozaril
1140910358	Chlorpromazine
1140867208	Perphenazine
1140909802	Levomepromazine
1140867134	Pericyazine
1140867306	Dolmatil
1140867210	Fentazin
1140867398	Fluphenazine
1140867078	Benperidol
1140867218	Pimozide
1141201792	Zaponex
1141200458	Denzapine
1140867136	Neulactil
1140879750	Thioridazine
1140867180	Dozic
1140867546	Fluspirilene
1140928260	Panadeine
1140927956	Sertindole
1140867490	Lithium product
1140867494	Camcolit 250 tablet
1140867498	Liskonum 450mg m/r tablet
1140867500	Phasal 300mg m/r tablet
1140867504	Priadel 200mg m/r tablet
1140867518	Litarex 564mg m/r tablet
1140867520	Li-liquid 5.4mmol/5ml oral solution

Note: Adapted from Davis et al. (2019), doi: 10.1002/mpr.1796. *medication code not included in Davis et al. (2019).
