# Supplementary material for: Lifetime depression and age-related changes in body composition, cardiovascular function, grip strength and lung function: sex-specific analyses in the UK Biobank
Source: Aging (Albany NY). 2021 Jul 7;13(13):17038–79. doi: 10.18632/aging.203275 (PMC8312429; doi:10.18632/aging.203275)
Supplement: Supplementary Material 5 [file aging-13-203275-s005.pdf]

Supplementary Material 5. Density plots physiological measures.

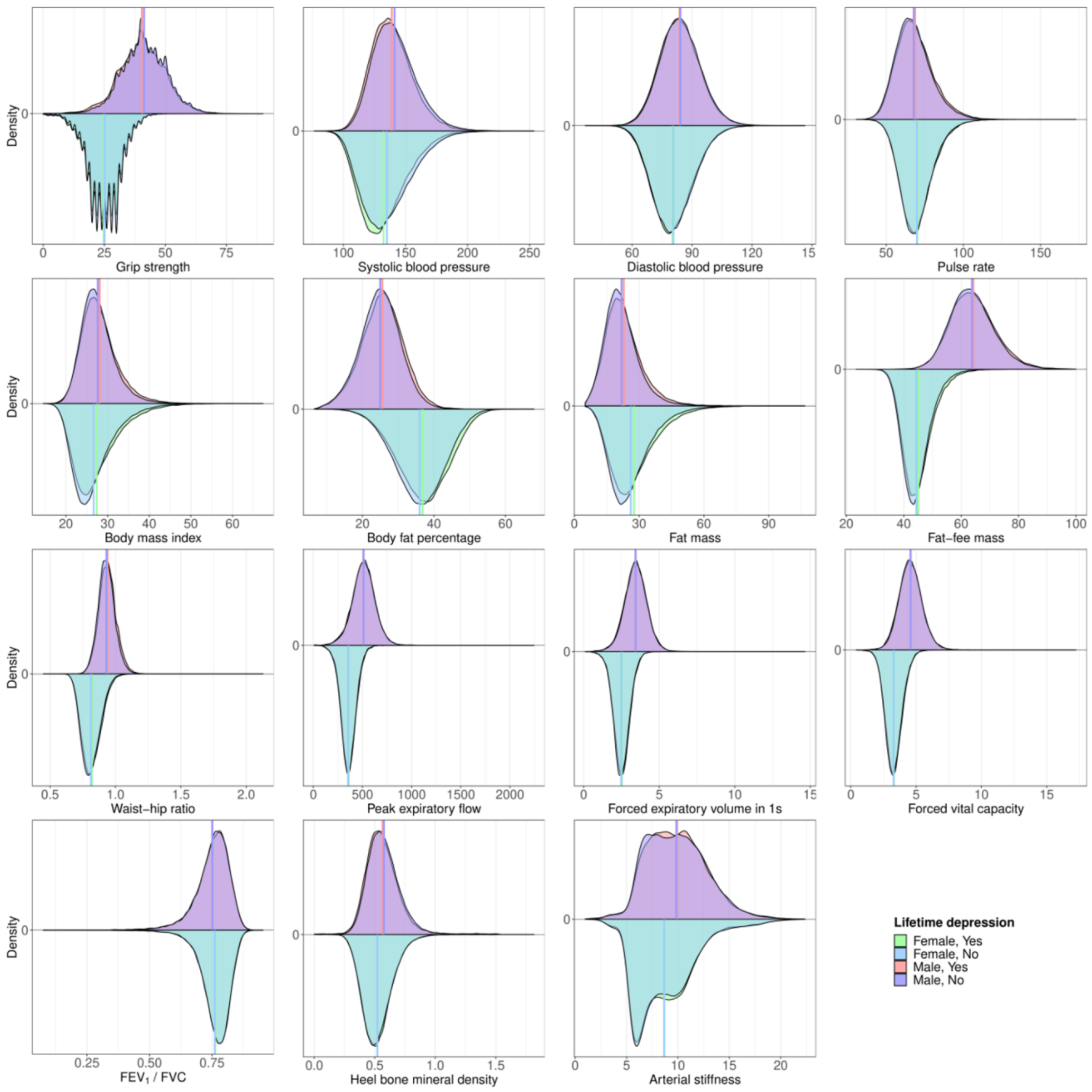

Supplementary Figure 1. Physiological measures of males and females with lifetime depression and healthy controls. Arterial stiffness truncated at 99.9th %ile. FEV_1, forced expiratory volume in one second; FVC, forced vital capacity.
