# Supplementary material for: Lifetime depression and age-related changes in body composition, cardiovascular function, grip strength and lung function: sex-specific analyses in the UK Biobank
Source: Aging (Albany NY). 2021 Jul 7;13(13):17038–79. doi: 10.18632/aging.203275 (PMC8312429; doi:10.18632/aging.203275)
Supplement: Supplementary Material 6 [file aging-13-203275-s006.pdf]

Supplementary Material 6. Sample characteristics.

Supplementary Table 3. Sample characteristics.

	Overall (N=502521)	Female		Male	
		Healthy control (N=123842)	Depression (N=56276)	Healthy control (N=131212)	Depression (N=31063)
**Age**					
Mean (SD)	56.53 (8.10)	55.90 (8.03)	54.74 (7.78)	56.37 (8.26)	55.71 (7.96)
**Ethnicity**					
White	472711 (94.1%)	117587 (94.9%)	54386 (96.6%)	125053 (95.3%)	30089 (96.9%)
Mixed-race	2958 (0.6%)	805 (0.7%)	434 (0.8%)	588 (0.4%)	145 (0.5%)
Asian	8061 (1.6%)	2096 (1.7%)	556 (1.0%)	1779 (1.4%)	195 (0.6%)
Black	9882 (2.0%)	1809 (1.5%)	459 (0.8%)	2518 (1.9%)	393 (1.3%)
Chinese	1574 (0.3%)	527 (0.4%)	91 (0.2%)	357 (0.3%)	37 (0.1%)
Other	4558 (0.9%)	1018 (0.8%)	350 (0.6%)	917 (0.7%)	204 (0.7%)
Prefer not to answer	1662 (0.3%)				
Do not know	217 (<0.1%)				
Missing	898 (0.2%)				
**Household income**[1]					
Very low	97205 (19.3%)	26243 (21.2%)	13986 (24.9%)	21903 (16.7%)	7181 (23.1%)
Low	108177 (21.5%)	32464 (26.2%)	14751 (26.2%)	31452 (24.0%)	7472 (24.1%)
Middle	110774 (22.0%)	32735 (26.4%)	14575 (25.9%)	36631 (27.9%)	8233 (26.5%)
High	86269 (17.2%)	25352 (20.5%)	10605 (18.8%)	31999 (24.4%)	6599 (21.2%)
Very high	22930 (4.6%)	7048 (5.7%)	2359 (4.2%)	9227 (7.0%)	1578 (5.1%)
Prefer not to answer	49848 (9.9%)				
Do not know	21305 (4.2%)				
Missing	6013 (1.2%)				
**Walking**[2]					
Mean (SD)	5.39 (1.93)	5.48 (1.84)	5.35 (1.95)	5.31 (1.99)	5.23 (2.06)
Prefer not to answer	979 (0.2%)				
Unable to walk	1929 (0.4%)				
Do not know	6687 (1.3%)				
Missing	874 (0.2%)				
**Moderate activity**[2]					
Mean (SD)	3.63 (2.33)	3.62 (2.31)	3.51 (2.34)	3.59 (2.31)	3.46 (2.35)
Prefer not to answer	2273 (0.5%)				
Do not know	24120 (4.8%)				
Missing	878 (0.2%)				
**Vigorous activity**[2]					
Mean (SD)	1.84 (1.96)	1.73 (1.85)	1.61 (1.82)	2.10 (2.02)	1.95 (2.02)
Prefer not to answer	4116 (0.8%)				
Do not know	22582 (4.5%)				
Missing	878 (0.2%)				
**Smoking status**					
Never	273528 (54.4%)	77069 (62.2%)	30307 (53.9%)	67927 (51.8%)	13959 (44.9%)
Former	173064 (34.4%)	37682 (30.4%)	19633 (34.9%)	49044 (37.4%)	12473 (40.2%)
Current	52979 (10.5%)	9091 (7.3%)	6336 (11.3%)	14241 (10.9%)	4631 (14.9%)
Prefer not to answer	2059 (0.4%)				
Missing	891 (0.2%)				
**Alcohol intake frequency**					
Never	40645 (8.1%)	9217 (7.4%)	4658 (8.3%)	6290 (4.8%)	2323 (7.5%)
Special occasions	58011 (11.5%)	16310 (13.2%)	8361 (14.9%)	8259 (6.3%)	2436 (7.8%)
1-3/month	55856 (11.1%)	15862 (12.8%)	7869 (14.0%)	11184 (8.5%)	3054 (9.8%)
1-2/week	129294 (25.7%)	33072 (26.7%)	14017 (24.9%)	34139 (26.0%)	7516 (24.2%)
3-4/week	115443 (23.0%)	28093 (22.7%)	11628 (20.7%)	36606 (27.9%)	7587 (24.4%)
Daily/almost daily	101770 (20.3%)	21288 (17.2%)	9743 (17.3%)	34734 (26.5%)	8147 (26.2%)

Prefer not to answer	605 (0.1%)				
Missing	897 (0.2%)				
**Sleep duration**					
Mean (SD)	7.15 (1.11)	7.18 (1.00)	7.17 (1.17)	7.13 (0.99)	7.11 (1.21)
Prefer not to answer	386 (0.1%)				
Do not know	2943 (0.6%)				
Missing	887 (0.2%)				

Note: Descriptive statistics for covariates based on main dataset *N*=342,393. [1]Annual household income groups: very low (<£18,000), low (£18,000–30,999), middle (£31,000–51,999), high (£52,000–100,000) and very high (>£100 000). [2]number of days per week engaging in these activities for 10+ minutes continuously.
