# Supplementary material for: Lifetime depression and age-related changes in body composition, cardiovascular function, grip strength and lung function: sex-specific analyses in the UK Biobank
Source: Aging (Albany NY). 2021 Jul 7;13(13):17038–79. doi: 10.18632/aging.203275 (PMC8312429; doi:10.18632/aging.203275)
Supplement: Supplementary Material 7 [file aging-13-203275-s007.pdf]

Supplementary Material 7. Adjusted GAMs.

7A. Age-related changes in females.

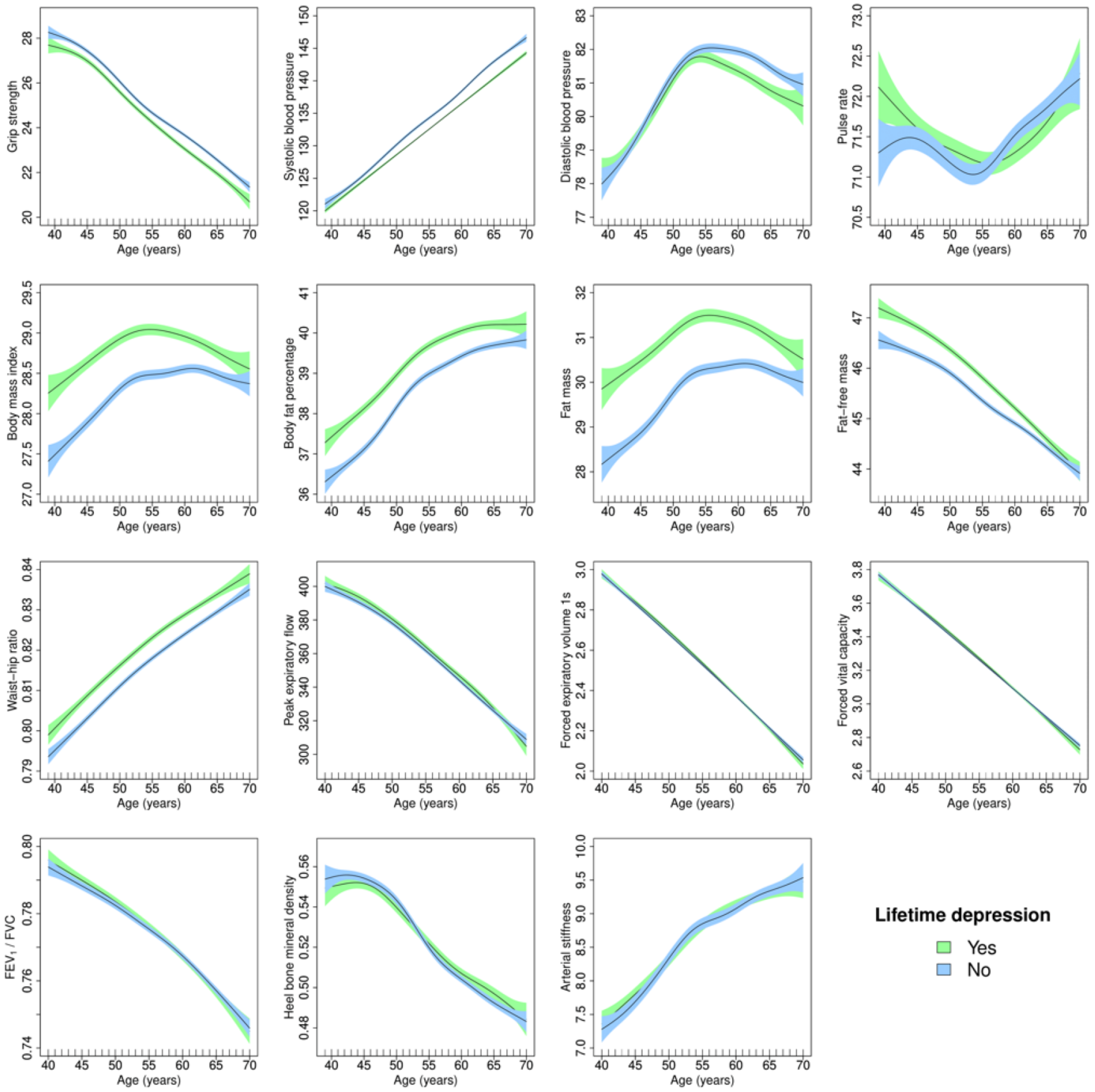

Supplementary Figure 2. Adjusted generalised additive models of age-related changes in physiological measures in females with lifetime depression and healthy controls. Models were adjusted for ethnicity (except lung function), gross annual household income, physical activity, smoking status, alcohol intake frequency, sleep duration and, for cardiovascular measures, current use of antihypertensive medications. The solid lines represent physiological measures against smoothing functions of age. The shaded areas correspond to approximate 95% confidence intervals (± 2 × standard error). FEV_1, forced expiratory volume in one second; FVC, forced vital capacity.

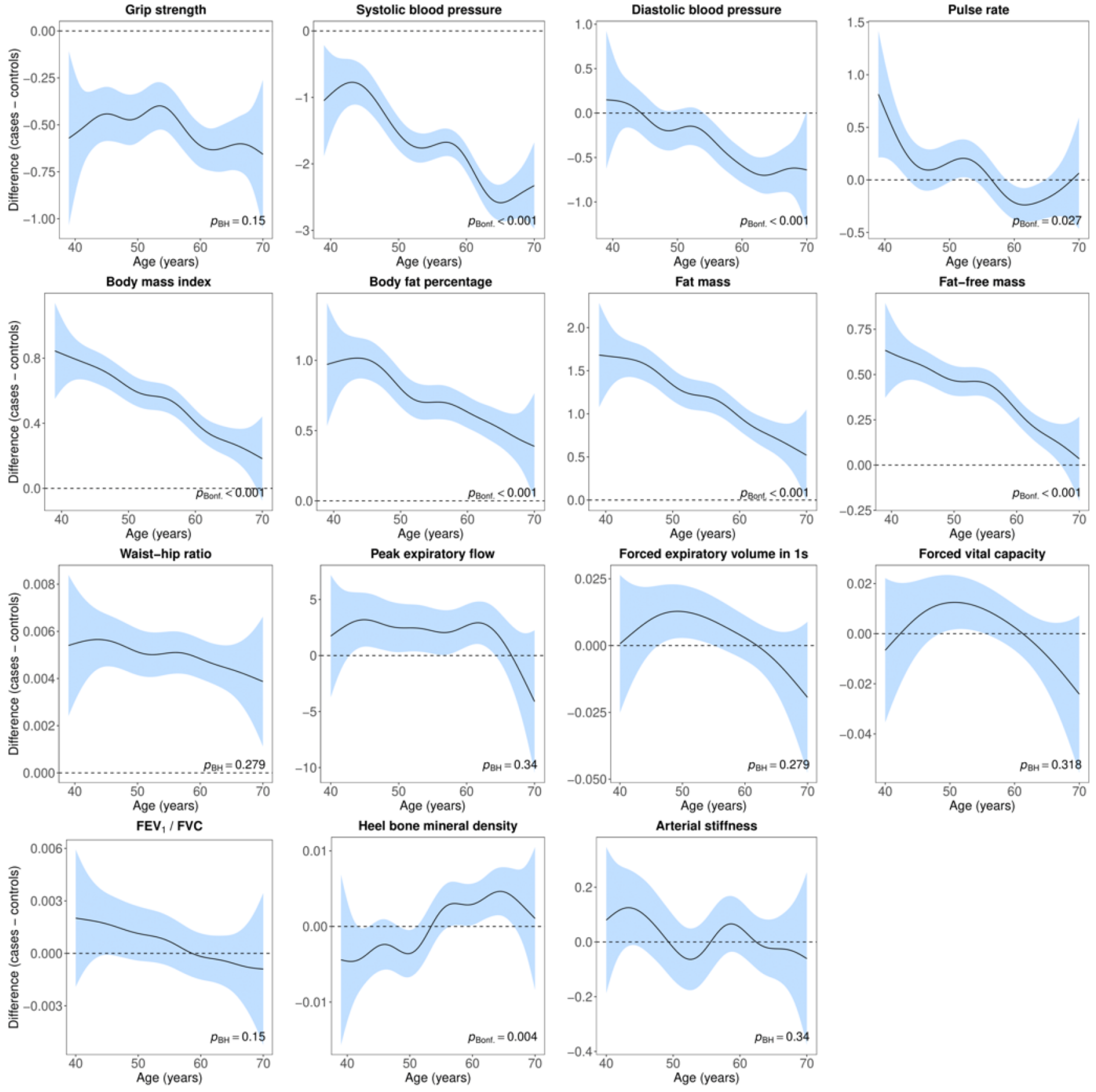

Supplementary Figure 3. Difference smooths comparing age-related changes in physiological measures of females with lifetime depression to healthy controls. Models were adjusted for ethnicity (except lung function), gross annual household income, physical activity, smoking status, alcohol intake frequency, sleep duration and, for cardiovascular measures, current use of antihypertensive medications. The shaded areas correspond to approximate 95% confidence intervals (± 2 × standard error). Negative values on the y-axes correspond to lower values in females with lifetime depression compared to healthy controls. The horizontal lines represent no difference between female cases and controls. FEV_1, forced expiratory volume in one second; FVC, forced vital capacity; Bonf, Bonferroni; BH, Benjamini and Hochberg.

7B. Age-related changes in males.

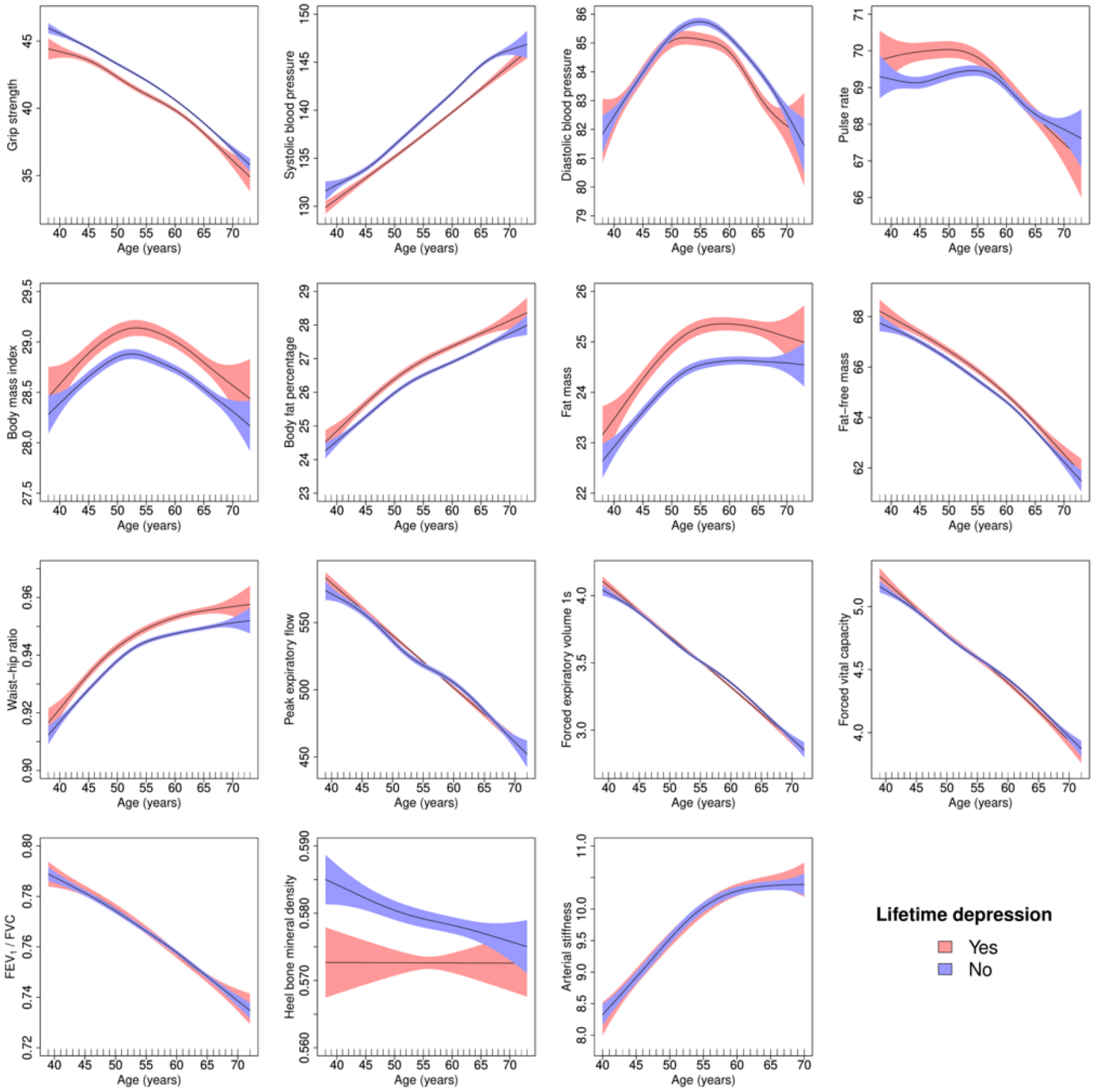

Supplementary Figure 4. Adjusted generalised additive models of age-related changes in physiological measures in males with lifetime depression and healthy controls. Models were adjusted for ethnicity (except lung function), gross annual household income, physical activity, smoking status, alcohol intake frequency, sleep duration and, for cardiovascular measures, current use of antihypertensive medications. The solid lines represent physiological measures against smoothing functions of age. The shaded areas correspond to approximate 95% confidence intervals (± 2 × standard error). FEV_1, forced expiratory volume in one second; FVC, forced vital capacity.

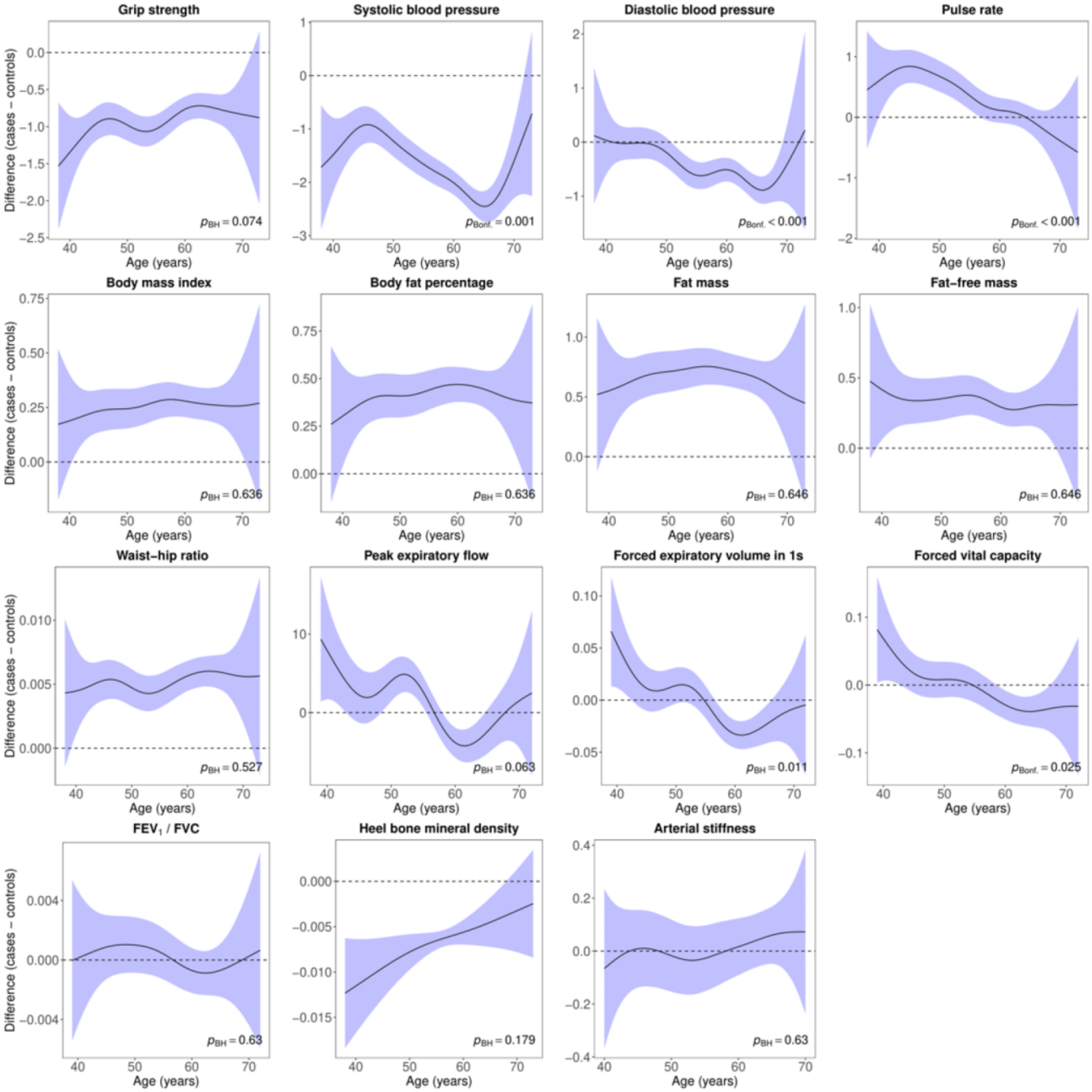

Supplementary Figure 5. Difference smooths comparing age-related changes in physiological measures of males with lifetime depression to healthy controls. Models were adjusted for ethnicity (except lung function), gross annual household income, physical activity, smoking status, alcohol intake frequency, sleep duration and, for cardiovascular measures, current use of antihypertensive medications. The shaded areas correspond to approximate 95% confidence intervals (± 2 × standard error). Negative values on the y-axes correspond to lower values in males with lifetime depression compared to healthy controls. The horizontal lines represent no difference between male cases and controls. FEV_1, forced expiratory volume in one second; FVC, forced vital capacity; Bonf, Bonferroni; BH, Benjamini and Hochberg.
