# Supplementary material for: Lifetime depression and age-related changes in body composition, cardiovascular function, grip strength and lung function: sex-specific analyses in the UK Biobank
Source: Aging (Albany NY). 2021 Jul 7;13(13):17038–79. doi: 10.18632/aging.203275 (PMC8312429; doi:10.18632/aging.203275)
Supplement: Supplementary Material 8 [file aging-13-203275-s008.pdf]

Supplementary Material 8. Sensitivity analyses.

8A. Case-control numbers.

Supplementary Table 4. Case-control numbers sensitivity analyses.

Dataset	Sex	≥ 2 Depression measures		CIDI-SF lifetime depression (MHQ)	
		Healthy control	Depression	Healthy control	Depression
Main dataset	Female	123842	23517	30113	20482
	Male	131212	11785	32776	9432
Lung function	Female	43133	8010	10750	7162
	Male	52359	4617	13586	3826
Bone mineral density	Female	85994	12219	19420	12907
	Male	89790	6075	21289	6005
Arterial stiffness	Female	38592	11525	10818	7688
	Male	42889	5899	11805	3528

Note: CIDI-SF, Composite International Diagnostic Interview Short Form; MHQ, mental health questionnaire.

8B. Case control differences.

Supplementary Table 5. Differences in physiological measures between individuals with lifetime depression and healthy controls.

Variable	≥ 2 depression measures					CIDI-SF lifetime depression (MHQ)				
	SMD	95% CI		$p_{Bonf.}$	p_{BH}	SMD	95% CI		$p_{Bonf.}$	p_{BH}
**Female**										
Hand-grip strength	-0.067	-0.081	-0.053	<0.001	<0.001	-0.042	-0.060	-0.025	<0.001	<0.001
Systolic blood pressure	-0.192	-0.206	-0.178	<0.001	<0.001	-0.162	-0.180	-0.145	<0.001	<0.001
Diastolic blood pressure	-0.047	-0.061	-0.033	<0.001	<0.001	-0.005	-0.023	0.013	>0.999	0.600
Pulse rate	0.028	0.014	0.042	0.002	<0.001	0.052	0.034	0.070	<0.001	<0.001
Body mass index	0.190	0.176	0.204	<0.001	<0.001	0.230	0.212	0.248	<0.001	<0.001
Body fat percentage	0.165	0.151	0.179	<0.001	<0.001	0.193	0.175	0.210	<0.001	<0.001
Fat mass	0.211	0.197	0.225	<0.001	<0.001	0.227	0.209	0.245	<0.001	<0.001
Fat-free mass	0.181	0.167	0.195	<0.001	<0.001	0.166	0.148	0.184	<0.001	<0.001
Waist-hip ratio	0.115	0.101	0.129	<0.001	<0.001	0.122	0.104	0.139	<0.001	<0.001
Peak expiratory flow	0.120	0.096	0.144	<0.001	<0.001	0.073	0.043	0.103	<0.001	<0.001
Forced expiratory volume 1s	0.110	0.086	0.134	<0.001	<0.001	0.067	0.037	0.097	<0.001	<0.001
Forced vital capacity	0.102	0.078	0.126	<0.001	<0.001	0.049	0.019	0.079	0.021	0.002
FEV_1 / FVC	0.043	0.019	0.067	0.006	<0.001	0.051	0.021	0.081	0.011	0.001
Heel bone mineral density	0.024	0.005	0.043	0.177	0.013	0.028	0.005	0.050	0.227	0.017
Arterial stiffness	0.007	-0.014	0.028	>0.999	0.448	0.016	-0.013	0.045	>0.999	0.301
**Male**										
Hand-grip strength	-0.116	-0.135	-0.097	<0.001	<0.001	-0.002	-0.025	0.020	>0.999	0.900
Systolic blood pressure	-0.159	-0.178	-0.140	<0.001	<0.001	-0.143	-0.166	-0.120	<0.001	<0.001
Diastolic blood pressure	-0.044	-0.063	-0.025	<0.001	<0.001	-0.002	-0.025	0.021	>0.999	0.900
Pulse rate	0.109	0.090	0.128	<0.001	<0.001	0.088	0.066	0.111	<0.001	<0.001
Body mass index	0.140	0.121	0.158	<0.001	<0.001	0.192	0.169	0.215	<0.001	<0.001
Body fat percentage	0.134	0.115	0.153	<0.001	<0.001	0.142	0.119	0.165	<0.001	<0.001
Fat mass	0.165	0.146	0.184	<0.001	<0.001	0.183	0.160	0.206	<0.001	<0.001
Fat-free mass	0.097	0.078	0.116	<0.001	<0.001	0.137	0.114	0.160	<0.001	<0.001
Waist-hip ratio	0.167	0.148	0.185	<0.001	<0.001	0.167	0.144	0.190	<0.001	<0.001
Peak expiratory flow	0.005	-0.025	0.035	>0.999	0.793	0.019	-0.017	0.055	>0.999	0.413
Forced expiratory volume 1s	0.003	-0.027	0.033	>0.999	0.837	0.045	0.009	0.081	0.224	0.022
Forced vital capacity	0.011	-0.019	0.041	>0.999	0.564	0.046	0.011	0.082	0.190	0.021
FEV_1 / FVC	-0.013	-0.043	0.017	>0.999	0.517	0.006	-0.030	0.042	>0.999	0.900
Heel bone mineral density	-0.101	-0.127	-0.075	<0.001	<0.001	-0.074	-0.102	-0.045	<0.001	<0.001
Arterial stiffness	0.020	-0.007	0.047	>0.999	0.092	0.001	-0.037	0.038	>0.999	0.974

Note: SMD, standardised mean difference; CI, confidence interval; Bonf, Bonferroni; BH, Benjamini and Hochberg; FEV_1, forced expiratory volume in one second; FVC, forced vital capacity; CIDI-SF, Composite International Diagnostic Interview Short Form; MHQ, mental health questionnaire. *P*-values for Welch's t-test.

8C. Age-related changes in females.

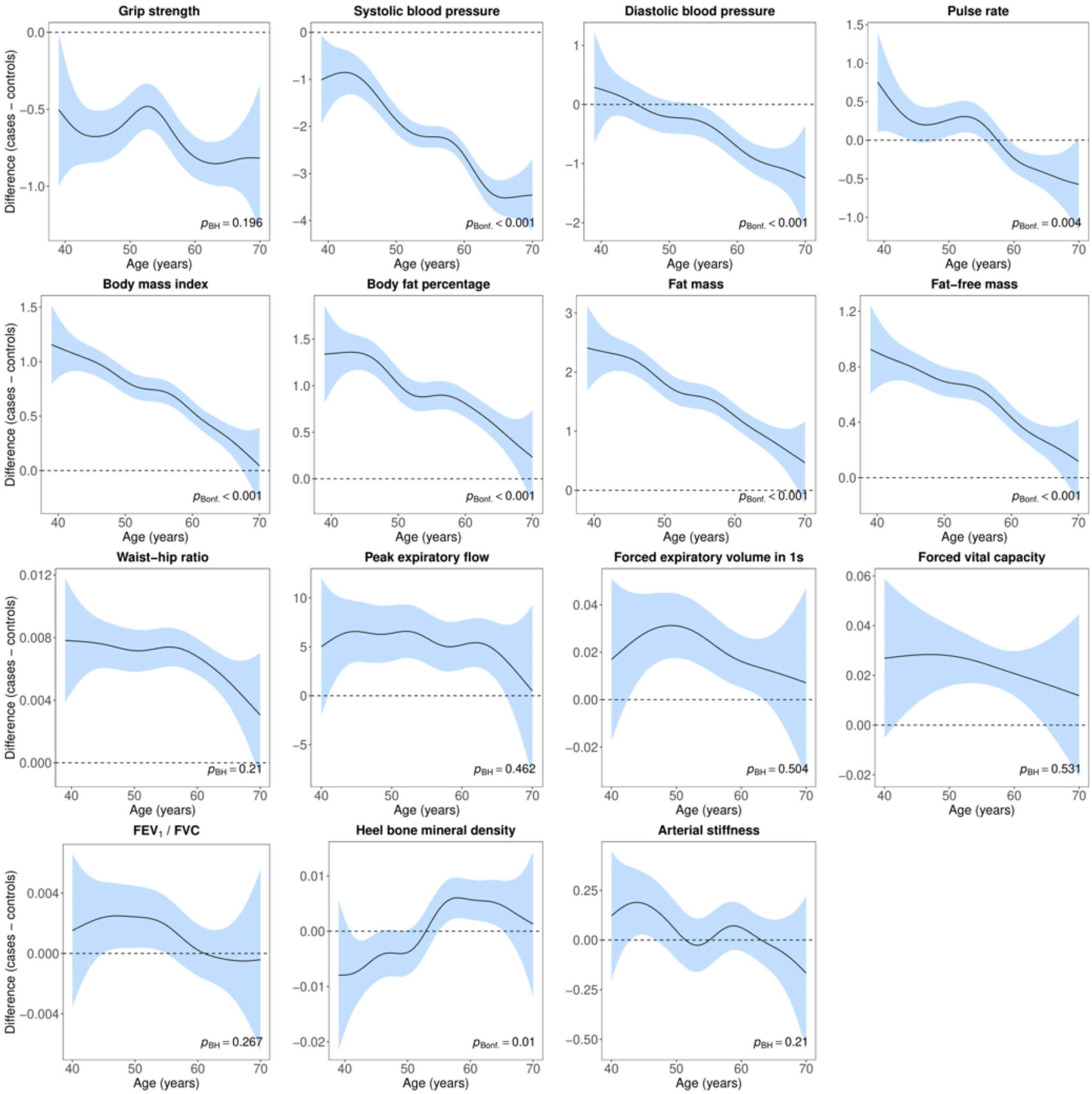

Supplementary Figure 6. Difference smooths comparing age-related changes in physiological measures of females with lifetime depression (≥ 2 depression measures) to healthy controls. Models were adjusted for ethnicity (except lung function), gross annual household income, physical activity, smoking status, alcohol intake frequency, sleep duration and, for cardiovascular measures, current use of antihypertensive medications. The shaded areas correspond to approximate 95% confidence intervals (± 2 × standard error). Negative values on the y-axes correspond to lower values in females with lifetime depression compared to healthy controls. The horizontal lines represent no difference between female cases and controls. FEV_1, forced expiratory volume in one second; FVC, forced vital capacity; Bonf, Bonferroni; BH, Benjamini and Hochberg.

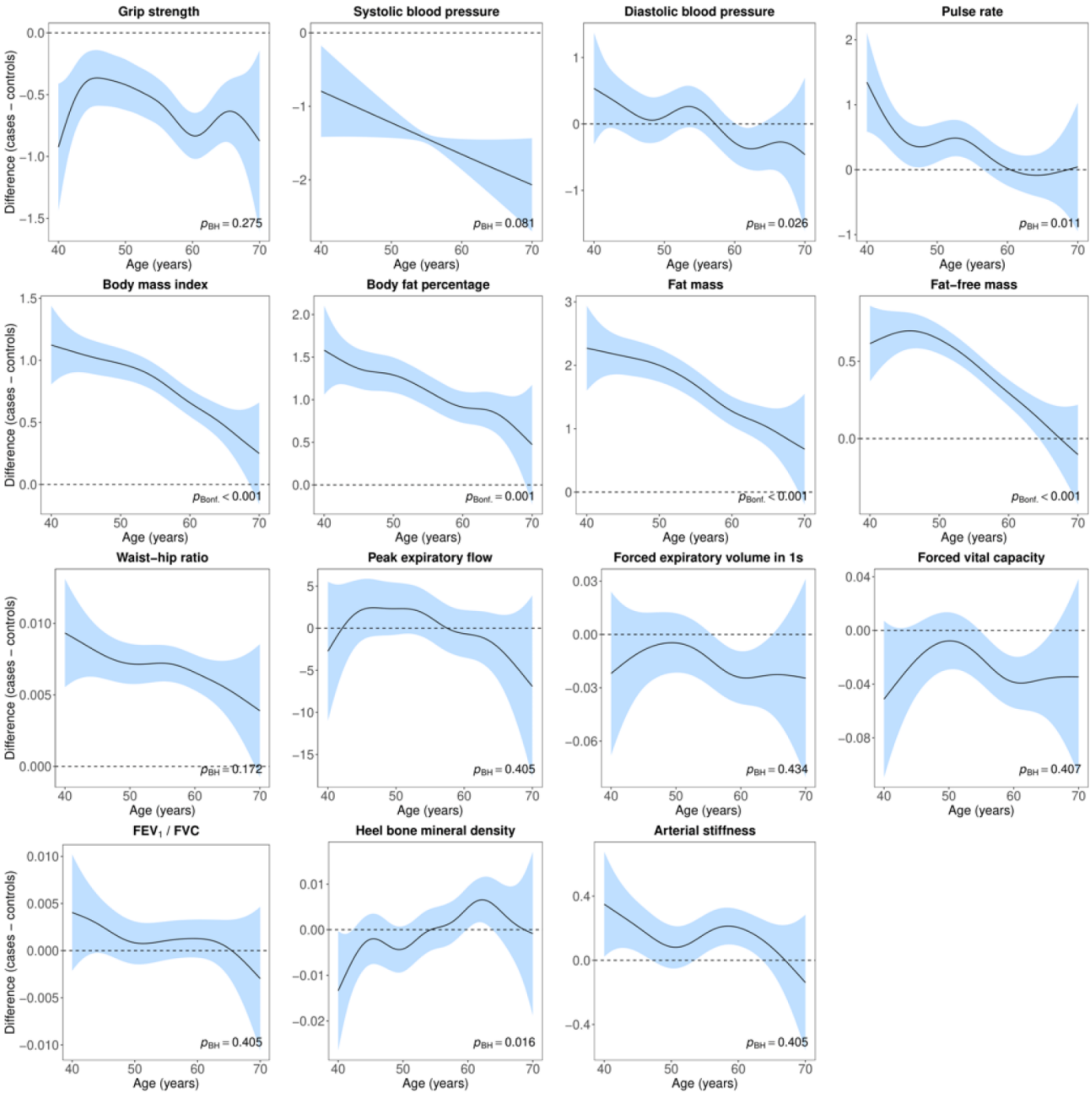

Supplementary Figure 7. Difference smooths comparing age-related changes in physiological measures of females with lifetime depression (CIDI-SF) to healthy controls. Models were adjusted for ethnicity (except lung function), gross annual household income, physical activity, smoking status, alcohol intake frequency, sleep duration and, for cardiovascular measures, current use of antihypertensive medications. The shaded areas correspond to approximate 95% confidence intervals (± 2 × standard error). Negative values on the y-axes correspond to lower values in females with lifetime depression compared to healthy controls. The horizontal lines represent no difference between female cases and controls. CIDI-SF, Composite International Diagnostic Interview Short Form; FEV_1, forced expiratory volume in one second; FVC, forced vital capacity; Bonf, Bonferroni; BH, Benjamini and Hochberg.

8D. Age-related changes in males.

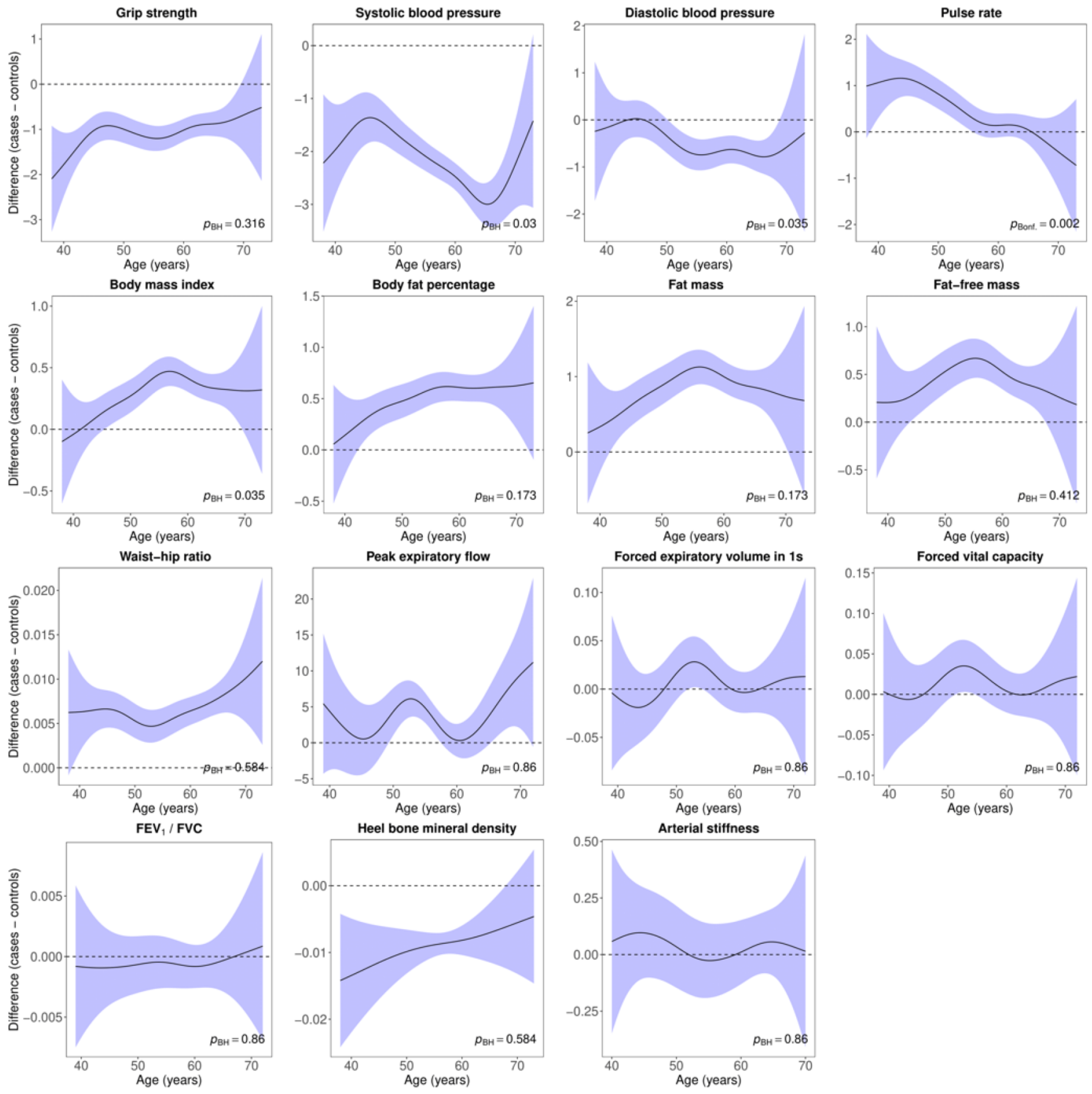

Supplementary Figure 8. Difference smooths comparing age-related changes in physiological measures of males with lifetime depression (≥ 2 depression measures) to healthy controls. Models were adjusted for ethnicity (except lung function), gross annual household income, physical activity, smoking status, alcohol intake frequency, sleep duration and, for cardiovascular measures, current use of antihypertensive medications. The shaded areas correspond to approximate 95% confidence intervals (± 2 × standard error). Negative values on the y-axes correspond to lower values in males with lifetime depression compared to healthy controls. The horizontal lines represent no difference between male cases and controls. FEV_1, forced expiratory volume in one second; FVC, forced vital capacity; Bonf, Bonferroni; BH, Benjamini and Hochberg.

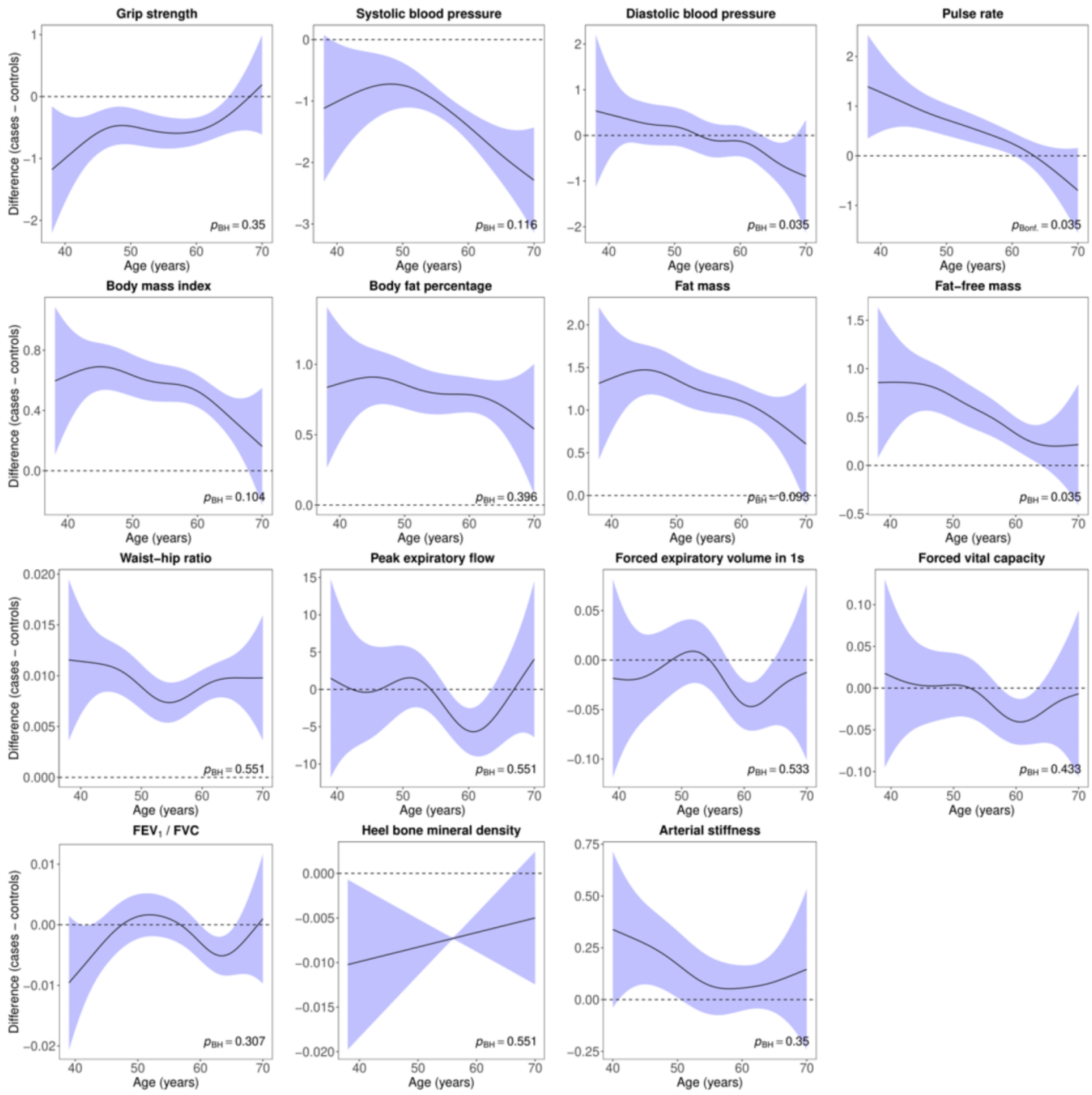

Supplementary Figure 9. Difference smooths comparing age-related changes in physiological measures of males with lifetime depression (CIDI-SF) to healthy controls. Models were adjusted for ethnicity (except lung function), gross annual household income, physical activity, smoking status, alcohol intake frequency, sleep duration and, for cardiovascular measures, current use of antihypertensive medications. The shaded areas correspond to approximate 95% confidence intervals (± 2 × standard error). Negative values on the y-axes correspond to lower values in males with lifetime depression compared to healthy controls. The horizontal lines represent no difference between male cases and controls. CIDI-SF, Composite International Diagnostic Interview Short Form; FEV_1, forced expiratory volume in one second; FVC, forced vital capacity; Bonf, Bonferroni; BH, Benjamini and Hochberg.
